# Outcomes of the conservative management of the patients with endometrial intraepithelial neoplasia/endometrial cancer: Wait or treat!

**DOI:** 10.55730/1300-0144.5385

**Published:** 2022-02-05

**Authors:** Esra İŞÇİ BOSTANCI, Yasin DURMUŞ, A. Sinem DURU ÇÖTELİ, Fulya KAYIKÇIOĞLU, Nurettin BORAN

**Affiliations:** 1 The department of Gynecologic Oncology, Faculty of Medicine, Gazi University, Ankara, Turkey; 2 The department of Gynecologic Oncology, Mersin City Training and Research Hospital, Mersin, Turkey; 3 The department of Gynecologic Oncology, Etlik Zübeyde Hanım Women’s Health Training and Research Hospital, Ankara, Turkey

**Response to letter to the Editor**

It is a great honor for us to have this letter to the editor for our manuscript. Therefore, we would like to thank Dr. Iavazzo and Dr. Gkegkes for their comments on our study.

They have mentioned in their letter; Corderio et al. [1] reported that the uterine cavity was evaluated via hysteroscopy (53/54 of patients) and intrauterine synechiae were detected in 10 patients. In our study, the major cavity control procedures were probe curettage and dilatation & curettage, only 6 of all patients were evaluated by hysteroscopy. In this group of patients that were evaluated via hysteroscopy, there was no sign of intrauterine synechiae. Therefore, we were not able to present the rate of intrauterine synechiae formation in our manuscript.

In our series, there were no diabetic patients or history of metformin use so we could not make any comment about metformin combination/DM (diabetes mellitus) correlation with fertility outcome.

According to our data, the mean value of the BMI was 32.85 kg/m^2^ (range 20–48) and 65.8% of the patients (n = 25) have BMI of 30 kg/m^2^ or greater. In this group, there were only two pregnancies and one live birth. When we analyzed the data, a weak significance was found between BMI and lower pregnancy rates (p = 0.046). The Fisher’s exact test, where appropriate, was used for comparison, and no significance was found (p = 0.071).

To sum up, we have appreciated this letter and we hope this manuscript would be a reference for the upcoming research about this topic.
